# Seasonality of Litter Insects and Relationship with Rainfall in a Wet Evergreen Forest in South Western Ghats

**DOI:** 10.1673/031.009.4601

**Published:** 2009-06-24

**Authors:** Anto Anu, Thomas K Sabu, PJ Vineesh

**Affiliations:** Litter Entomology Research Unit, St. Joseph's College, Devagiri, Calicut, Kerala-673008, India

## Abstract

The seasonality of litter insect abundance and its relationship with rainfall was analyzed in a wet evergreen forest on the windward side of south Western Ghats. Monthly litter samples were collected using Berlese funnels during 4 seasons of a year: southwest monsoon season June—August), northeast monsoon season (September—November), summer (March -May) and pre-summer season (December—February). Insect fauna as a whole showed no seasonal variation in abundance, however, some individual insect orders showed significant seasonal variation. Overall insect fauna and individual orders were distributed independently relative to rainfall. All insect orders with the exception of Psocoptera were present during all four seasons. Coleoptera (42%) was the dominant group in all seasons followed by Formicidae (12.3%), insect larvae (10.1%), Collembola (9.2%) and Thysanoptera (8.9%). Exceptionally high abundance of Ptiliidae contributed to the unprecedented abundance of litter Coleoptera. The aseasonality of litter insect fauna as a whole is attributed to year-round availability of rainfall and the absence of severe summer conditions.

## Introduction

Seasonal variation in abundance of tropical insects is a common phenomenon (Wolda 1988; Pinheiro et al. 2002). A sharp reduction in abundance during the dry season seems to be restricted to tropical habitats that have a severe dry season (Janzen and Schoener 1968; Janzen 1973a.janzen 1973b; Wolda 1977). Conversely, several groups of insects are known to decline in number during the wet mid-season, sometimes exhibiting an abundance that is lower than that observed during the dry season (Robinson and Robinson 1970; Boinski and Fowler 1989; Pinheiro et al. 2002). On average, activity patterns of tropical species tend to be longer, the percentage of species active around the year higher, and the seasonal peaks less well defined relative to temperate insects (Wolda 1988). Though various macroclimatic and microclimatic changes (temperature, photoperiod, rainfall, humidity, decomposition rates of litter) and variation in the availability of food resources are the important factors in triggering seasonal activity of insects in tropical regions (Tauber and Tauber 1976; Denlinger 1986; Wolda 1988; Basset 1991; Tanaka 2000; Kai and Corlet 2002; Pinheiro et al. 2002; Nahrung and Allen 2004; Nakamura and Numata 2006; Anu 2006; Danks 2006; Vineesh 2007), however, the onset of rain is the major factor (Levings and Windsor 1982, Levings and Windsor 1985; Lowman 1982; Wolda and Denlinger 1984; Wolda 1988; Boinski and Fowler 1989; Frith and Frith 1990; Sabu et al. 2008). Nevertheless, the above inferences are generally based on the analysis of seasonal distributional patterns and life history of predominantly arboreal insects, as very limited data exists on the abundance of litter insects across seasons from tropical rain forest systems. Three basic types of litter insect seasonality patterns have been documented from tropical regions: 1) reduction in abundance during the wet season of Barro Colorado Island, Panama (Levings and Windsor 1985), 2) a peak in abundance during the wet season of North Queensland, Australia (Frith and Frith 1990), Atlantic coastal forest, south-east Brazil (Develey and Peres 2000), East Arc Mountains, Tanzania (Burgess et al. 1999), Tambopata Reserve, south-east Peru (Pearson and Derr 1986), Kibale Forest, Western Uganda (Nummellin 1989) and 3) a general aseasonality in Corcovado, Costa Rica (Boinski and Fowler 1989).

The direct effect of rainfall arises from the physical effects of large amounts of water falling on litter fauna and forest floor litter (Chiba et al. 1975; Boinski and Fowler 1989). Indirectly, rainfall affects the severity of the dry season, and the quantity of litter falling to the forest floor (Swamy and Proctor 1994; Clark et al. 2003; Wood et al. 2005; Sundarapandian et al. 2005). However, quantitative studies investigating the seasonal variability of litter insect abundance and its link with rainfall are lacking from the tropical rain forests in the Western Ghats, a global hot spot of biodiversity and the only tropical forest ecoregion of the Indian Peninsula (Myers et al. 2000; WWF 2001) that is well known for regional variation in vegetation, rainfall patterns and topography (Nair 1991; Patwardhan and Asnani 2000). Here we present comprehensive data on the abundance and the seasonal variability of leaf litter insect fauna of a wet evergreen forest in the Wayanad region of south Western Ghats that will providevaluable baseline data for future research.

## Materials and Methods

### Study site and climate

**Figure 1.**
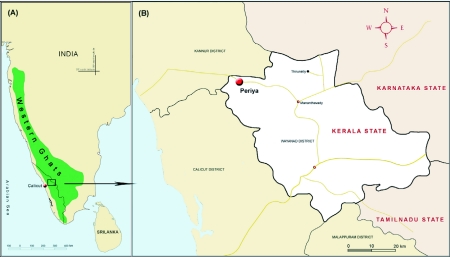
(A) Map of south Western India showing the location of the Western Ghats and (B) study site in the Wayanad region of the Western Ghats

**Figure 2.**
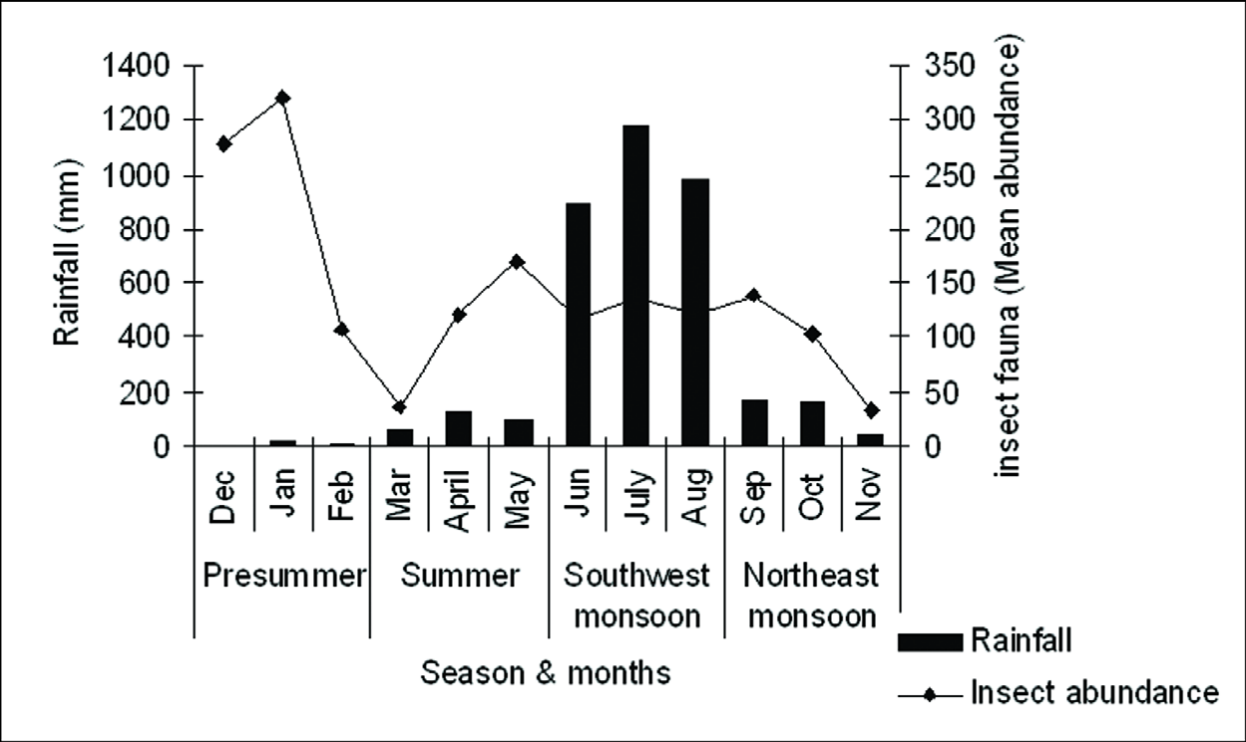
Rainfall received and litter insect abundance in the evergreen habitat during the study period 2002–03.

The study site is located in Periya (an evergreen forest patch covering an area of 85.12 sq km) on the western slope of the North Wayanad Western Ghats ecoregion (11°50′ N latitude and 75°49′ E longitude) (Figure 1). Forests in the region are medium elevation evergreen forests (Barboni and Bonnefille 2001) at an altitude of 800–850 above sea level. Southwest and the northeast monsoons control the climate of the region. An annual rainfall of 3752 mm occurred during the study period (2002–03), of which, 81% occurred during the southwest monsoon season June—August), 10% during the northeast monsoon season (September—November), 8% during the summer (March—May) the remaining 1% during the pre-summer season (December—February) (Figure 2). The southwest monsoon is the wettest season in the region (average monthly rainfall of 1013 mm) and rain is continuous during this period. Mostly afternoon showers occur during the northeast monsoon (average monthly rainfall of 128.7 mm). The hot and dry season extends from March to May with April and May being the hottest months with an average temperature of 31° C and occasional heavy rains occur (average monthly rainfall of 97.1 mm). The pre-summer season receives the lowest rains (average monthly rainfall of 11.3 mm).

Biogeographically, the Wayanad region of the Western Ghats is a transitional zone between the moist *Cullenia* sp. dominated forests of south Western Ghats and the dry dipterocarp forests of the northern region (Rodgers and Panwar 1988; WWF 2001; Wikramanayake et al. 2001). The Wayanad was once a swath of lush tropical evergreen forest that extended between the lowland Malabar coast evergreen forests in the west and the moist deciduous forests of Western Ghats in the east (WWF 2001; Nair 1991). But today, as a result of years of forest clearing to establish rubber, coffee, tea and teak plantations, human settlements and other human activities very little of the natural habitat is left. Therefore, the original evergreen character of the forests has changed to a semi-ever-green and deciduous condition (Champion and Seth 1968; Rodgers and Panwar 1988; Nair 1991). Currently, Wayanad is placed within the south Western Ghats moist deciduous forests ecoregion and the Periya is one among the very few blocks of old growth evergreen forest remaining in the region.

Mid-elevation evergreen forests of the Periya belong to the Cullenia-Mesua-Palaquium type (Pascal 1991; Barboni and Bonnefille 2001). The vegetation consists of two layers in addition to subordinate shrub and herb layers, with the abundance of epiphytes in the interior regions. A litter layer of 4–7 cm thickness covers the forest floor.

### Data collection

Litter samples were collected for 12 months from December 2002 to November 2003, inclusive. Samples were taken at random along a 25 m grid line during the course of the last 8 days of each month covering all four seasons. Three samples of litter were collected per month for a total of nine samples per season and 36 samples in total. The mean of the nine seasonal samples was used to evaluate the abundance of insect fauna for a particular season. The time of sampling was between 0830 hrs and 1030 hrs. During the rainy season, sampling was done three days after the rain to minimize the direct effect of rain on various fauna (Burgess et al. 1999). During June and July, it was not possible to strictly adhere to this pattern and sampling was done on any non-rainy day in the last week of the month. Plots were located in areas of minimum human disturbance (i.e. 500 m away from the road).

Each sample was collected by placing a 1/4 m^2^ (50 × 50 cm) wooden quadrat frame on the forest floor and then scraping up all litter and loose humus from within the framed area into a large polythene bag (Frith and Frith 1990). Samples were collected as quickly as possible to prevent animals from escaping. The polythene bag was securely tied and returned to the laboratory. The litter collected for analysis refers to the upper organic litter plus the loose humus layer. No underlying compact soil was taken.

Each sample was placed in a series of 15–20 cm diameter Berlese funnels fitted with 4–6 mm mesh screen and a 60 watt light bulb for 36 hrs and the invertebrates were collected in 70% alcohol in a conical flask. Animals too large to be extracted by this method were removed visually. Extracted fauna was sorted and categorized up to order level and major fauna (Coleoptera and Collembola) up to family level. Larval forms were collectively categorized as insect larvae, as the smaller size of the soft bodied forms and the deformation which occurred during the Berlese funnel extraction makes further categorization and grouping unfeasible. Groups with a mean abundance of >50 individuals per sample and present during all seasons were considered major groups. Insect orders with a mean abundance of 15–50 individuals per sample were considered minor groups and <15 were regarded as insignificant marginal fauna.

### Data analysis

Distribution of litter insects among samples was non-normal. Hence, non-parametric statistics were employed for data analysis. Multivariate comparisons were done using Kruskal-Wallis H tests (Sachs 1992), to evaluate the significance level of seasonal differences in insect abundance, followed by a Mann-Whitney U test to determine seasonal differences in individual orders. The relation between insect abundance and rainfall was analyzed using non-parametric linear regression analysis. In this distribution free method, the linear relationship between total rainfall (mm) received during the entire month (predictor, independent), and the total insect abundance and individual insect orders of the corresponding month (dependant, outcome) is investigated. For all analysis, significance was determined at *P*<0.05. Statistical analysis was done with MegaStat Version 10.0 (Orris 2005) and StatsDirect statistical software version 2.6. The Bray-Curtis similarity index (Bray and Curtis 1957) was used to compare the similarity between the insect communities from different seasons followed by hierarchical agglomerative Cluster analysis using Primer v5.2.9 (Clarke and Gorley 2002).

## Results

### Overall abundance and seasonality

A total of twelve litter insect orders were collected over the four seasons (Table 1). No significant seasonal pattern was found in the overall abundance (i.e. all orders) of litter insect fauna (H = 3.30, df = 3, *P*>0.05). However, there was a trend towards higher insect fauna during pre-summer (42% of the total faunal abundance), and low insect fauna abundance during the northeast monsoon period (17% of the total faunal abundance) (Figure 2). With the exception of Psocoptera, recorded only during the summer and southwest monsoon seasons, members of all other insect orders were present during all seasons. Coleoptera (42%) was the dominant group in all seasons followed by Formicidae (12.3%), insect larvae (10.1%), Collembola (9.2%) and Thysanoptera (8.9%). These five groups collectively accounted for 82 % of the total individuals captured. Psocoptera (4.04%) and Isoptera (5.9%) were the prominent minor insect groups. Blattaria (1.52%), Dermaptera (0.27%), Diptera (1.39%), Hemiptera (1.66%) and Orthoptera (1.97%) were present in very low numbers. Among the major groups, Coleoptera with 14 families was the most diverse group. Ptiliidae (30.2 ± 29.3) and Staphylinidae (21.2 — 20.1) were the most abundant coleopteran families during all seasons. Dolichoderinae was the dominant ant family and Entomobryidae (8.0 — 7.2) the dominant collembolan family.

### Seasonality of major and minor groups

Eight out of the 12 insect orders showed seasonal variability. Three insect orders *viz*., Hemiptera, Dermaptera, Diptera, and insect larvae, did not show significant seasonal variation in abundance (*P* >0.05) (Table 1). Four broad patterns were distinct in the seasonal distribution of fauna. Coleoptera, Formicidae and Collembola were significantly higher during pre-summer; Thysanoptera, Isoptera and Psocoptera peaked during summer; Orthoptera peaked during northeast monsoon period (Table 1 and 2). During the pre-summer season, Coleoptera contributed >50 % of the insect faunal abundance. The response of Coleoptera was mostly driven by Ptiliidae, which represented over 41% of all individual beetles collected. Staphylinidae were the dominantColeoptera during summer and Ptiliidae during all other seasons. Collembola recorded a bimodal peak in abundance in pre-summer and in southwest monsoon season. The abundance of Thysanoptera was significantly higher during the summer and pre-summer season. Isoptera peaked during summer and recorded lowest abundance during southwest and northeast monsoon seasons.

**Table 1.**
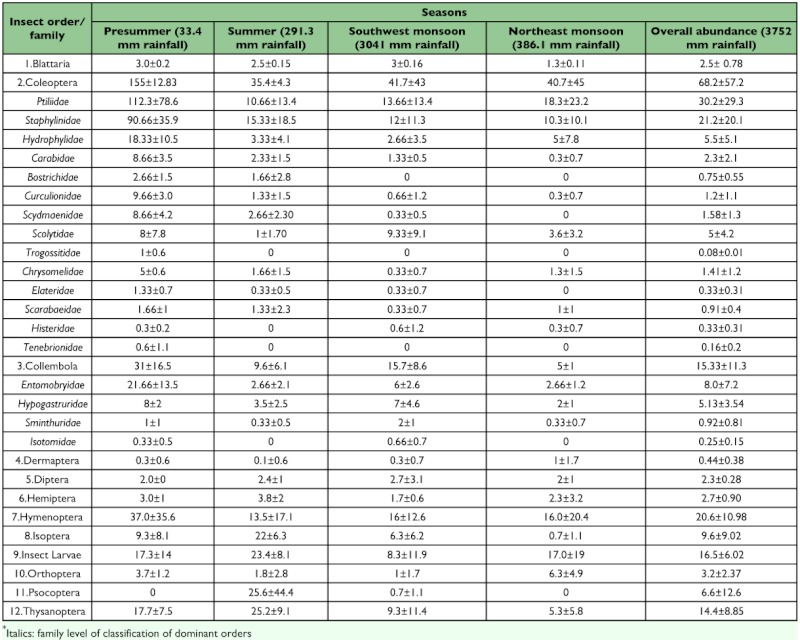
Insect numbers (mean ± SE) per 1/4 m2 (50 × 50 cm) litter samples (n=9 for seasons and n=36 for overall abundance) at Periya during 2002–03 study period.

**Figure 3.**
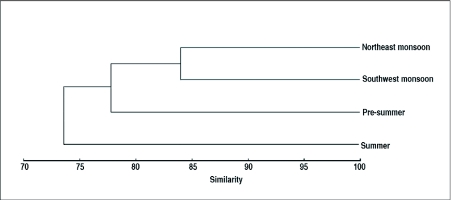
Dendrogram based on hierarchical agglomerative clustering (group-linking) of litter insect faunal assemblage at Periya during 4 different seasons of the study period 2002–03.

Based on faunal similarity among seasons, two broad clusters were identified, *viz*., rainy season and non-rainy season fauna. The greatest faunal similarity was observed during the southwest and northeast monsoon seasons, combined in the dendrogram at a similarity level of 83.96%. Summer fauna showed the greatest dissimilarity from the rest (Figure 3).

### Precipitation and fauna

Non-parametric linear regression between rainfall and overall faunal abundance were not significant (*P*>0.05).

**Table 2.**
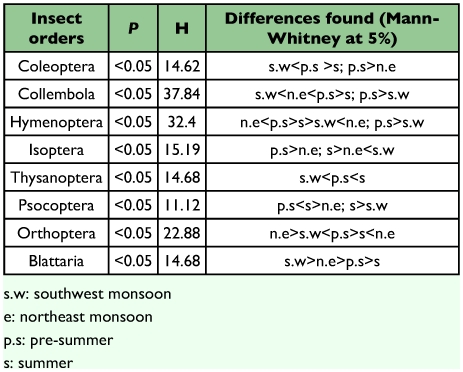
Results of Kruskal Wallis and Mann-Whitney tests on the seasonal variation of individual insect orders during the study period at Periya.

## Discussion

### Seasonality of fauna

Contrary to expectations, the abundance of total litter insect fauna did not vary among seasons in this mid-elevation tropical evergreen forest. Although the abundance of insects did not vary seasonally, the individual insect groups that comprised the population did. Though these patterns were quantified for only a single year in this study, casual observations made in earlier studies (Sabu 2005) support the observed findings. Fluctuations in rainfall appeared to have no role in deciding the abundance of individual and overall fauna. In view of other variables such as litter depth, litter moisture, humidity and temperature that are directly or indirectly related to rainfall and litter insect population densities (Wagner et al. 2003; Lensing et al. 2005; Vineesh et al. 2007), these findings considering the influence of rainfall alone must be examined with caution.

### Comparison of seasonality with other litter fauna studies

The aseasonality of litter insect abundance observed in the region deviate from the distinct seasonality in other regions (Frith and Frith 1990; Burgess et al. 1999) where abundance of fauna peaked during wet period and declined during the dry summer period. We attribute this to the occurrence of rainfall during all seasons and the presence of moderate summer conditions in the study region. Both Australia and Tanzania (Frith and Frith 1990; Burgess et al. 1999) received 50% less rainfall during wetter months compared to Western Ghats. In both of these regions the excessively wet period lasts for one month and the summer is longer and harsh with very low faunal abundance. In contrast, the study region in the Western Ghats experiences a short duration summer of 3 months with short spells of heavy summer rains followed by 3 months of excessively wet months and another 3 months of moderate rains. Altogether continuous rainfall exists in the region for 6 months. We relate the more even presence of litter fauna at all times to round the year availability of rainfall in the region and the absence of harsh dry summer conditions both of which might result in the presence of uniform litter microhabitat conditions for fauna.

### Fauna in our collections

Fauna in our collections are typical of the insect fauna recorded from other tropical evergreen forests. Collembola, insect larvae, Formicidae and Coleoptera are the major fauna as in all earlier tropical rain forest litter fauna studies. The abundance relationship Coleoptera > Formicidae > insect larvae > Collembola > Thysanoptera was unlike the relationship Collembola > Formicidae > Coleoptera > Orthoptera > Psocoptera found in Africa (Burgess et al. 1999) and Collembola > insect larvae > Formicidae > Diptera found in Australia (Frith and Frith 1990). Higher abundance of Coleoptera with their numbers surpassing even that of Collembola and Formicidae were not previously recorded. Our findings support the observations made by Frith and Frith (1990) that faunal abundance relative to other groups vary between different geographical regions. Overall densities of litter insect fauna in our collections were higher compared to other tropical evergreen forests (Frith and Frith 1990).

### Relationship with rainfall

There was no significant relationship between rainfall and insect faunal abundance either as a whole group or individual insect groups. No earlier studies from tropical forests could establish a significant relationship between rainfall and insect faunal abundance, which should not be surprising, given the large number of groups involved and the many direct and indirect ways in which weather could separately influence the abundance of each group (Pinheiro et al. 2002). Analysis even at the family level taxonomic scale did not find a significant influence of rainfall in the abundance of major insect groups. These results suggest that analysis at the order level did not limit the capacity to predict the patterns. Though rainfall did not have a significant direct effect on faunal abundance in the region, there may be indirect effects, for example, by the influence of rainfall on plant phenology (litter fall and litter depth) and habitat microclimatic conditions (litter moisture, humidity conditions) (Levings and Windsor 1984; Boinski and Fowler 1989). Further studies considering the influences of these independent variables may enable better prediction of the relationship between fauna and physical factors.

### Abundance and seasonality of insect major groups Coleoptera

Predominance of Coleoptera as the major faunal element has not been recorded in studies of tropical rainforest litter fauna conducted elsewhere using Berlese funnels (Madge 1965; Holt 1985; Frith and Frith 1990; Burgess et al. 1999). The dominance of Coleoptera in the present study arises from the exceptionally higher abundance of Ptiliidae that prefer moist soil, litter and rotting wood, and are a potential bio-indicator of moist habitats (Hall 2001; Sawada and Hirowatari 2002; Sörensson 2003). Their negligible presence in the predominantly deciduous forests of the Wayanad region, highlights the conservation importance of this last remaining, climax wet evergreen forest in Wayanad region (Nair 1991; Sabu 2005; Anu 2006). The abundance of hydrophylids and staphylinid beetles, known for their preference for wet litter conditions are further evidence for this hypothesis (Borror et al. 1996; Lawrence 1999). The low abundance of Coleoptera in other evergreen forest studies raises two possibilities. Either it is a regional pattern peculiar to the region or it arises from the overlooking of Ptiliidae in litter samples in studies elsewhere as their small size (rarely > 1mm) and cryptic colors tend to limit their detection (Caesar 2004).

### Formicidae

The predominance of Formicidae as the second most abundant group in evergreen forest litter habitat was recorded earlier (Burgess et al. 1999). The presence of two genera that prefer wet rainforests, *Acropyga* sp. and *Paratrechina* sp. (Shattuck and Barnett 2001), not recorded from any other forest vegetation types in the region (Anu and Sabu 2006) is also evidence for the moist wet litter conditions in south Western Ghats. The peak in abundance of Formicidae during the rainless pre-summer is related to their low preference for wet litter habitat conditions which makes foraging difficult for litter Formicidae (Brühl et al. 1999). What causes their reduction in summer is not understood. Possibly the summer rains in April or a few days prior to our collection might have limited the foraging of Formicidae (Janzen 1973b; Brühl et al. 1999).

### Insect larva

Abundance of larvae was high throughout the study period in concurrence with the high larval abundance reported in the Australian evergreen forest (Frith and Frith 1990).

### Collembola

The lower abundance of Collembola is in stark contrast to their predominance in litter faunal studies in other tropical forest regions employing Berlese funnels (Madge 1965; Holt 1985; Burgess et al. 1999) and other forests in the region (Sabu 2005). What leads to the lower presence of these moist litter-preferring groups in the present study site is not understood. Their peak in population level in pre-summer could be linked to the well known migratory behaviour of Collembola to favourable litter moisture conditions following the monsoon period and the peak in southwest monsoon season also to the same behaviour when habitats becomes excessively wet (Lensing et al. 2005).

### Other insect groups

Thysanoptera, Pscocoptera and Isoptera peaked in the summer season. Similar high abundance of Thysanoptera has been reported only from certain Australian forests (Holt 1985). The summer dominance of Psocoptera was recorded from the wet forests of Panama (Levings and Windsor 1984). Although Isoptera are one of the most numerous tropical arthropods that generally forage widely on the forest floor (Wallwork 1976) most litter faunal studies including the present one (Madge 1965; Plowman 1979; Lasebikan 1988; Stork 1988) reported low numbers of Isoptera in litter/soil samples of tropical forests. Recent literature reveals that their cryptic nature, relative inactivity, range of nesting habits, feeding strategies and patchy distributions within and between habitats results in a low sampling efficiency with standard methods and a composite bait sampling study is necessary for effective termite collections (Abensperg-Traun and DeBoer 1990; Eggleton and Bignell 1995; Dawes-Gromadski 2005 a,b). Hence scarcity of Isoptera in the samples may be due to the sampling inefficiency of Berlese funnel methods to collect Isoptera. Very low incidence of Isoptera during the southwest and northeast monsoon periods are possibly linked to suppressed termite activity during saturated forest floor conditions (Dawes-Gromadski 2005 a,b).

## Conclusions

Our results provide the first glimpse of insect seasonality within the wet evergreen litter ecosystem of a regional forest in the Western Ghats. It is hoped that the results of this study will initiate further studies on litter insect seasonality in various forest types and localities in the windward and downwind regions of the Western Ghats, well known for regional variation in rainfall patterns and topography. Invertebrate seasonality patterns play an important role in regulating the feeding and breeding patterns of many tropical rain forest vertebrate species (Frith and Frith 1990). In this context, data on the seasonal component of litter insect abundance from the Western Ghats forests will be useful in the effort to understand the breeding, foraging ecology and distributional pattern of insectivorous vertebrate species in the region.
